# Tenofovir alafenamide fumarate attenuates bleomycin-induced pulmonary fibrosis by upregulating the NS5ATP9 and TGF-β1/Smad3 signaling pathway

**DOI:** 10.1186/s12931-019-1102-2

**Published:** 2019-07-22

**Authors:** Lingxia Li, Jing Zhao, Li Zhou, Jie Chen, Yuanyuan Ma, Yanyan Yu, Jun Cheng

**Affiliations:** 1 Department of Infectious Diseases, Peking University First Hospital, No.8 Xishiku Street, Xicheng District, Beijing, 100034 China; 2 Peking University Ditan Teaching Hospital, Beijing 100015, China; Beijing; Key Laboratory of Emerging Infectious Diseases, Beijing, 100015 China; 3 Department of Infectious Disease, China-Japan Friendship Hospital, Beijing, 100029 China; 4 Department of Infectious Disease, First Hospital of Shanxi Medical University, Taiyuan, 030001 China; 5 Laboratory Animal Center, Peking University First Hospital, Beijing, 100034 China; 6 Department of Infectious Diseases, Beijing Ditan Hospital, Capital Medical University, No.8 East Jingshun Street, Chaoyang District, Beijing, 100015 China

**Keywords:** Pulmonary fibrosis, NS5ATP9, Tenofovir alafenamide fumarate, Fibroblasts, Transforming growth factor-β1/Smad3 signaling pathway

## Abstract

**Background:**

Pulmonary fibrosis is a progressive and irreversible disease for which therapeutic options are currently limited. A recent in vivo study showed that tenofovir, a nucleotide analogue reverse transcriptase inhibitor, had direct antifibrotic effects on skin and liver fibrosis. Another study in vitro revealed that NS5ATP9 inhibited the activation of human hepatic stellate cells. Because of the similarity of fibrotic diseases, we hypothesized that tenofovir alafenamide fumarate (TAF), the prodrug of tenofovir, and NS5ATP9, is related to and plays a role in the suppression of pulmonary fibrosis.

**Methods:**

We investigated the influence of NS5ATP9 on fibrosis in vitro. Human lung fibroblasts (HFL1) were transfected with short interfering RNAs or overexpression plasmids of NS5ATP9 before stimulation by human recombinant transforming growth factor-β1. The effect of TAF was evaluated in a bleomycin-induced fibrosis murine model. Male C57BL/6 mice were treated with bleomycin on day 0 by intratracheal injection and intragastrically administered TAF or vehicle. Left lung sections were fixed for histological analysis, while homogenates of the right lung sections and HFL1 cells were analyzed by western blotting and quantitative reverse transcription polymerase chain reaction.

**Results:**

NS5ATP9 suppressed the activation of lung fibroblasts. Upregulation of collagen type 3 (α 1 chain) and α-smooth muscle actin was observed in HFL1 cells when NS5ATP9 was silenced, and vice-versa. TAF also showed anti-fibrotic effects in mice, as demonstrated by histological analysis of fibrosis and expression of extracellular matrix components in the lung sections. Additionally, TAF inhibited transforming growth factor-β1 and phosphorylated-Smad3 synthesis in HFL1 cells and the murine model, which was accompanied by upregulation of NS5ATP9.

**Conclusions:**

Our results suggest that NS5ATP9 forms a negative feedback pathway in pulmonary fibrosis and TAF has anti-fibrotic properties as it upregulates the expression level of NS5ATP9. As TAF has been shown to be safe and well-tolerated in humans, TAF and NS5ATP9 may be useful for developing novel therapeutics for pulmonary fibrosis.

**Electronic supplementary material:**

The online version of this article (10.1186/s12931-019-1102-2) contains supplementary material, which is available to authorized users.

## Background

Idiopathic pulmonary fibrosis (IPF) is a chronic, progressive, and fibrotic interstitial lung disease, with a five-year survival rate of less than 30% [1]. It is characterized by fibroblast proliferation and extracellular matrix (ECM) deposition, which eventually leads to abnormal remodeling of the lung tissue structure and death due to respiratory failure [2]. Although studies of the treatment of pulmonary fibrosis have been widely conducted, effective treatment options remain limited [3]. A classic animal model for studying the pathophysiology process associated with lung fibrosis is a bleomycin-induced mouse model [4]. In this model, a series of pathophysiology processes occur, such as recruitment of cytokines and inflammatory factors, proliferation of fibroblasts, excessive deposition of ECM, and participation and regulation of multiple signaling pathways [5, 6].

Over-synthesis of collagen is an essential process in pulmonary fibrosis. Collagen is synthesized by fibroblasts and myofibroblasts. Myofibroblasts, which are derived from fibroblasts, express α-smooth muscle actin (α-SMA) as well as fibronectin and mainly secrete collagen and other ECM components. Collagen type I and type III are the main collagen components involved in pulmonary fibrosis. Collagen type III, which is encoded by COL3A1, has been reported as an indicator of an early or active stage of pulmonary fibrosis [7, 8].

Transforming growth factor (TGF)-β1 is one of the most important fibrogenic cytokines in the development of pulmonary fibrosis. It not only triggers fibroblast differentiation into myofibroblasts, but also promotes collagen synthesis and excessive deposition of ECM [9–12]. SMAD proteins are downstream signaling molecules of TGF-β1 and involved in the regulation of signaling pathways in pulmonary fibrosis [13–15].

Hepatitis C virus nonstructural protein NS5A trans-regulated protein 9 (NS5ATP9), also known as KIAA0101, p15 (PAF), L5, and OEACT-1 (GenBank accession no. AF529370), is a proliferating cell nuclear antigen-binding protein that plays numerous functional roles in organisms, such as cell growth, differentiation, apoptosis, DNA duplication and repair, and signal transmission [16–18]. NS5ATP9 is also involved in the genesis and development of tumors, including pancreatic cancer, non-small cell lung cancer, anaplastic thyroid carcinoma, and liver cancer [19–24]. NS5ATP9 can attenuate liver fibrosis by inhibiting the activation of hepatic stellate cells, downregulating the TGF-β1/Smad3 signaling pathway, suppressing cell proliferation, and promoting apoptosis [26]. However, the antifibrotic effect of NS5ATP9 has never been evaluated in the lung; thus, we investigated the role of NS5ATP9 in pulmonary fibrosis.

The nucleoside reverse transcriptase inhibitor tenofovir alafenamide fumarate (TAF) is widely used to treat human immunodeficiency virus and hepatitis B virus (HBV) infections [25–27]. A 5-year open-label follow-up study aimed at assessing the effect of tenofovir showed that up to 5 years of treatment with tenofovir resulted in the regression of fibrosis and cirrhosis in patients with chronic HBV infection [28]. Another study in which tenofovir was delivered onto the skin of bleomycin-treated mice and liver of thioacetamide-treated mice suggested that tenofovir has a direct anti-fibrosis effect [29]. As a prodrug of tenofovir, TAF may also have antifibrotic effects in the liver. Fibrotic changes in different organs share common pathogenic pathways, including the recruitment of cytokines and inflammatory factors, proliferation of inflammatory cells and fibrogenic effector cells, excessive deposition of extracellular matrix, and participation in and regulation of multiple signaling pathways [30–32]. In this study, we evaluated the antifibrotic effect of TAF in a bleomycin-induced pulmonary fibrosis mice model and tried to investigated whether TAF modulated the TGF-β1/smsd3 profibrogenic pathways. Additionally, it was reported that a 1% gel of tenofovir potently induced NS5ATP9 expression after 7 days of treatment of vaginal epithelial cells from healthy women [33]. Furthermore, other in vitro studies also revealed that TAF was dynamic on cell line [34, 35]. Thus, we also investigated the effect and relationship of TAF and NS5ATP9 on human lung fibroblasts in vitro.

## Methods

### Animals

In this study, 75 seven-week-old male C57BL/6 mice weighing 25–30 g were used for the experiments. They were purchased from Beijing Vital River Laboratory Animal Technology Co., Ltd. (Beijing, China), and maintained in a controlled environment in the Laboratory Animal Center of Peking University First Hospital, Beijing, China, for 1 week at 22 °C with a 12-h day-night cycle before use. The animals were provided with standard recipe fodder and water ad libitum during the experimental period. All animal experiments were reviewed and approved by the Institutional Animal Care and Use Committee of Peking University First Hospital (Approval ID: J201722), and strictly conducted in compliance with their guidelines.

### Surgery and treatments

The mice were anesthetized with Avertin/ 2,2,2-Tribromoethanol (Sigma-Aldrich; 0.5 ml in 39.5 ml of saline i.p.); 60 (30 for treatment groups and another for model groups) were treated with 50 μL bleomycin (BLM; Selleck) so that the final concentration was 2 mg/kg, and the other 15 with 50 μL of saline (referred to as nonfibrotic negative controls) on day 0, both delivered by intratracheal injection. Following BLM intratracheal instillation, the 30 mice from treatment groups were further randomly divided into three groups, which were treated with TAF (Anhui Biochem Unitedpharmaceutical Co., LTD and Nanjing Chia Tai Tianqing Pharmaceutical Co., LTD) at a final concentration of 5.125 mg/kg on day 0 (the first group), day 7 (the second group) and day 14 (the third group) for a total of 3 weeks through administration by gavage once a day. Mice from the BLM and control groups were received an equal volume of saline. Mice from the treatment groups were sacrificed with a lethal dose of Avertin on day 21 (the first group), 28 (the second group) and 35 (the third group) according to the initial therapeutic point with TAF, so as the BLM and control animals. The left lung sections were excised and fixed by immersion in 4% paraformaldehyde in for histological analysis. The right lung sections were weighed, quickly frozen, and stored at − 80 °C.

### Histopathological analysis

Tissue samples from the left lungs, which were fixed with 4% paraformaldehyde were embedded with paraffin wax and subjected to routine processing. Serial paraffin sections (4 μm) were prepared using a rotator microtome, and deparaffinized tissue sections were stained with hematoxylin and eosin (H&E, Sigma-Aldrich, St. Louis, MO, USA) to evaluate morphological changes in the lungs or with Masson’s trichrome stain (Trichrome Stain Kit, Sigma-Aldrich) and picrosirius red stain (Picro-Sirius Red Stain Kit, ab150681, Abcam, Cambridge, UK) to evaluate the collagen content and distribution. Images were obtained using an Axio Imager 2 microscope (Carl Zeiss AG, Oberkochen, Germany) at 400× magnification and Axiocam 506 color (Carl Zeiss AG). Collagen was quantified using ImageJ software (NIH, Bethesda, MD, USA).

### Cell culture

Human lung fibroblasts (HFL1; Cell Resource Center of Shanghai Institute of Life Sciences, Chinese Academy of Sciences, Shanghai, China) were grown in Ham’s F12K (Kaighn’s; Gibco, Grand Island, NY, USA), containing 10% fetal bovine serum (Gibco) and 1% penicillin/streptomycin (Life Technologies, Carlsbad, CA, USA) in 6-well plates (Thermo Fisher Scientific, Waltham, MA, USA) at 37 °C and 5% CO2 at a density of 2 × 105 cells/well with 100% humidity. The medium was changed every 3 days. The cells were cultured to the third passage before being used in our study. Human recombinant TGF-β1 (5 ng/mL) was added to serum-starved cells to induce myofibroblast differentiation. After 24 h, TAF or SD-208 was added for 24 h. Next, the cells were lysed for quantitative reverse transcription polymerase chain reaction (RT-qPCR) and western blot analysis.

### Cell viability assay

Cell viability was determined using a CCK8 (Cell Counting Kit-8)-based in vitro toxicology assay (Sigma-Aldrich), which detects viable cells based on the production of yellow formazan dye. HFL1 cells were initially seeded into 96-well plates at a density of 2 × 104 cells/well and then incubated at 37 °C for 4 h. Cell culture media were replaced with complete media containing different concentrations of TAF and incubated for 24 or 48 h. After incubation, 10 μL of CCK8 was added to each well and the plates were incubated at 37 °C for 4 h. Absorbance was read at 450 nm using a Multiskan MK3 multi-mode microplate reader (THERMO Varioskan Flash, Thermo Scientific, USA). Cell viability (%) was calculated as the ratio of surviving cells between the TAF-treated group and control group. All treated samples and controls were tested in triplicate.

### Plasmid construction and transfection with siRNA

The human NS5ATP9 gene was amplified and inserted into the expression vector pcDNA3.1/Myc-His (−); short interfering (siRNA) duplexes against human NS5ATP9, as well as a negative control siRNA, were purchased from Invitrogen (Carlsbad, CA, USA) and used according to the manufacturer’s instructions. All the methods mentioned above have been previously described by Quan et al [18]

### RNA isolation and RT-qPCR

Total RNAs from lung tissues were isolated by using Trizol reagent (Invitrogen) and HFL1 cells by using Total RNA Kit I (Cat. #: R6834; Omega Bio-Tek) according to the protocol provided by the manufacturer. The isolated RNA was reverse-transcribed in a solution with a final volume of 20 μL containing oligo dT primer, dNTP, and reverse transcriptase (Prime Script™ RT reagent Kit, Cat. #: R037A; TaKaRa). Sequences of the mouse and human gene-specific primers used in this study were listed in Additional file 1: Tables S1.1 and S1.2. Quantitative Real-time PCR was performed with Power SYBR® Green PCR Master Mix (ABI, USA) as the instructions. The PCR analysis used the Applied Biosystems® 7500 Real-Time PCR Systems (ABI, USA), and the PCR data and the relative gene expression level in a particular sample was evaluated by 2-△△CT method. Fold-expression changes were calculated based on at least 3 replicates per group.

### Western blot analysis

Proteins were extracted from resuspending cells or lung tissues in RIPA lysis buffer (50 mM/L Tris-HCl with a pH of 7.40, 150 mM/L NaCl, 1% Triton-X-100, 0.5% sodium deoxycholate, 0.1% SDS, 1 mM/L EDTA; Thermo Scientific) containing a protease and phosphatase inhibitor cocktail (Roche). Equal amounts of protein were loaded and separated into 12% SDS-PAGE. Membranes were blocked with 5% defatted milk in TBS-T for 2 h at room temperature, followed by incubation for overnight in primary antibodies (listed in Additional file 1) diluted in blocking buffer at 4 °C, and incubation with horseradish peroxidase-conjugated secondary antibodies diluted in blocking buffer for 1.5 h at room temperature. Protein bands were detected using chemiluminescence reagents (Pierce® ECL Western Blotting Substrate, Thermo Scientific). For Western blot analysis of lung tissues, at least 5 mice were used for each group.

### Statistical analysis

All data were presented as the mean values ± standard error of the mean. Multiple groups were compared by one-way analysis of variance, followed by Bonferroni’s method. A *P* value < 0.05 was considered as statistically significant.

## Results

### NS5ATP9 suppresses the expression of collagen 3α1, α-SMA, and fibronectin in TGF-β1-stimulated HFL1 cells

**Fig. 1:**
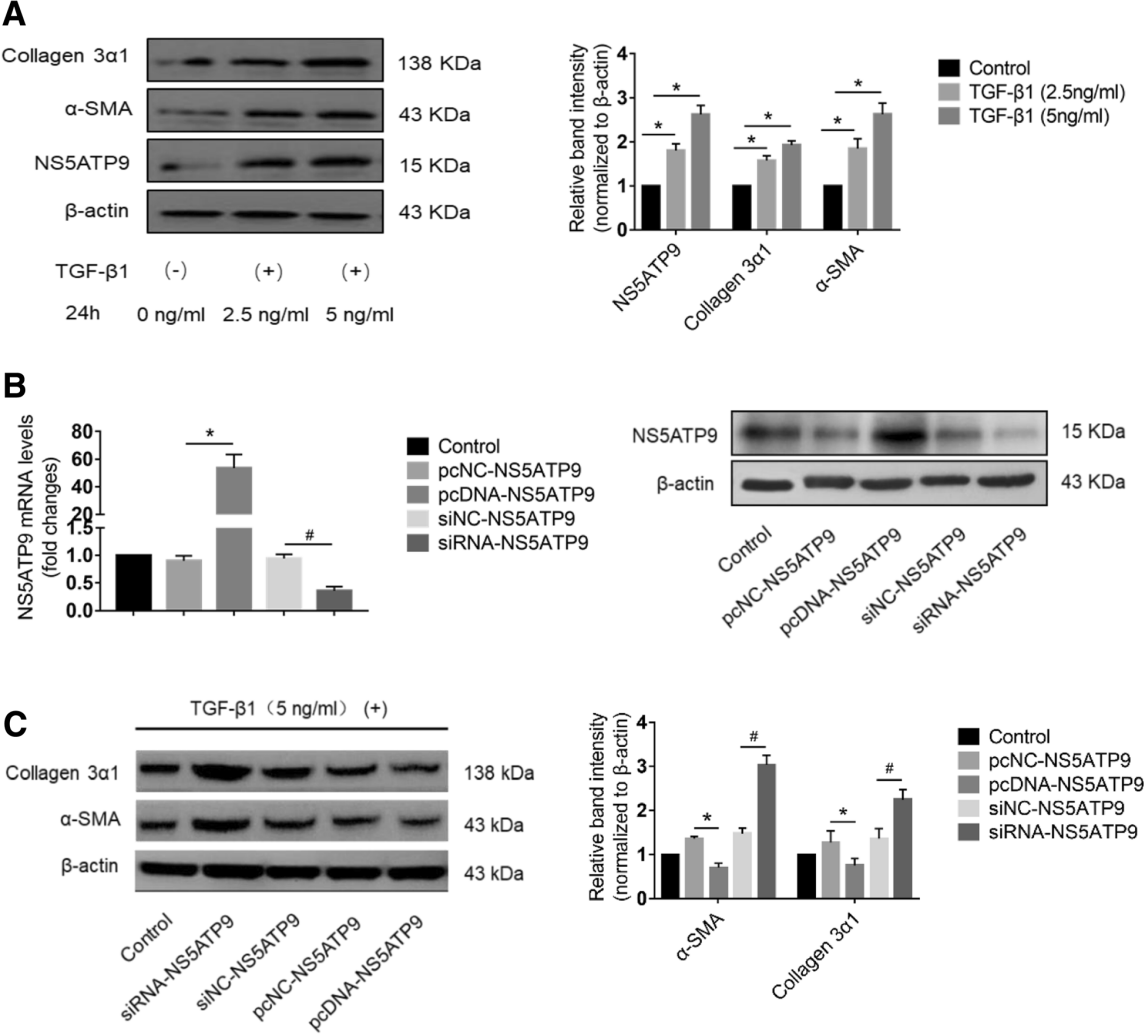
Expression level of NS5ATP9 in TGF-β1-induced HFL1 cells and its effects on suppressing ECM production. HFL1 cells were transfected transiently with pcDNA3.1/myc-His(−)-NS5ATP9 plasmid or siRNA-NS5ATP9 for 24 h. pcDNA3.1/myc-His(−) and siRNA-NC were used as their negative controls, respectively. Cells without transfection were used as the blank control. The expression level and inhibitory effects on ECM production of NS5ATP9 were analyzed by Western blotting (**a-c**), while the efficiency of RNA interference was evaluated at mRNA level via RT-qPCR (**b**). β-actin was used as an internal reference RT-qPCR. The expression level of proteins was evaluated by quantitative ratios shown as the relative optical densities of the bands normalized to β-actin expression. The results were shown as mean ± standard error of mean (SEM), and statistical significance across intervention groups was determined using one-way ANOVA. All assays were performed in triplicates. *P < 0.05 vs. pcNC-NS5ATP9 group, #*P* < 0.05 vs. siNC-NS5ATP9 group

The differentiation of fibroblasts into myofibroblasts stimulated by TGF-β1 is a key fibrogenic response in lung fibrosis [11, 12]. Therefore, we used human recombinant TGF-β1 protein to treat HFL1 cells, and then examined the expression level of NS5ATP9. We found that the protein level of NS5ATP9 was significantly increased after stimulation with TGF-β1 for 24 h in a dose-dependent manner (Fig. 1a). In addition, we observed similar upregulation in the expression levels of α-SMA, collagen 3α1, and fibronectin (Fig. 1a). Additionally, overexpression and silencing of NS5ATP9 were achieved in HFL1 cells. The plasmids of pcDNA3.1/myc-His (−)-NS5ATP9 (pcDNA-NS5ATP9) or negative control (pcDNA-NC) was transiently transfected into HFL1 cells. After 24 h, the overexpression of NS5ATP9 was successfully detected by RT-qPCR (Fig. 1b). Silencing of NS5ATP9 was verified by transient transfection of siRNA (siRNA-NS5ATP9) and control (siRNA-NC) (Fig. 1b).

To evaluate the effect of NS5ATP9 on HFL1 activation, HFL1 cells were stimulated with human recombinant TGF-β1 for 24 h after overexpression or silencing of NS5ATP9 for 24 h (Fig. 1c). We found that the protein levels of collagen 3α1 and α-SMA were downregulated when NS5ATP9 was overexpressed, while the opposite results were observed when NS5ATP9 was silenced (Fig. 1c).

### Effects of TAF on survival rate and body weight loss

**Fig. 2:**
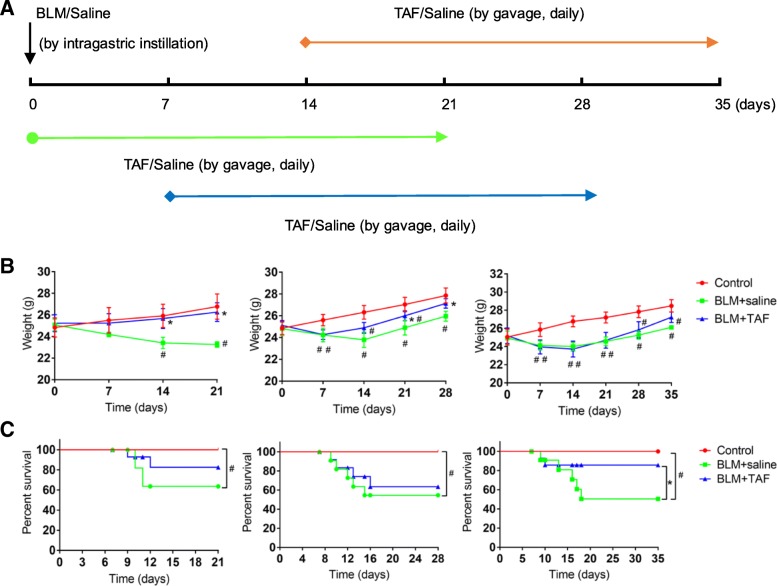
Protective effect of TAF on the BLM-injury mouse model. **a** Time course of BLM and TAF administration, as well as experimental termination. Mice were instilled intratracheally with BLM (2 mg/kg in 50 μl of saline). The treatment group received TAF (5.125 mg/kg in 50 μl of saline) intragastrically for 3 weeks separately on day 0, day 7, or day 14 after BLM instillation. The mice were sacrificed on day 21, day 28, or day 35 following BLM delivery, and lung tissues were collected for further study. **b** Body weight loss was lower in TAF group (BLM + TAF) than in BLM group (BLM + saline) within two weeks, and ponderal growth was higher in TAF group, compared to BLM group from the third week. **c** Survival rate across different treatment group. Mice in control group were all survival, and survival rate was higher in TAF group than in BLM group. The results were shown as mean ± standard error of mean (SEM), and statistical significance across intervention groups was determined using one-way ANOVA. #*P* < 0.05 vs. control group (saline + saline). *P < 0.05 vs. BLM group (BLM + saline)

To investigate the effects of TAF on BLM-induced pulmonary fibrosis, the mice were intragastrically administered with TAF (5.125 mg/kg) or vehicle once per day for 3 weeks on days 0, 7, and 14 after BLM injection at a dose of 2 mg/kg (Fig. 2a). The mice treated with BLM began to die on day 9, which agreed with the results of previous studies [6, 36]. Weight loss was lighter in TAF group, compared to BLM group (Fig. 2b). The mortality rate was 40–50% on days 21, 28, and 35 in the different BLM groups; in comparison, the mortality rate was 0–20% in mice treated with TAF (Fig. 2c) during the treatment period. Additionally, compared to mice treated with TAF on days 7 and 14 after administration of BLM, the mice treated with TAF starting on day 0 showed significant improvement in their survival rate and body weight loss.

### TAF attenuates BLM-induced histopathological changes in a murine model of BLM-induced pulmonary fibrosis

**Fig. 3:**
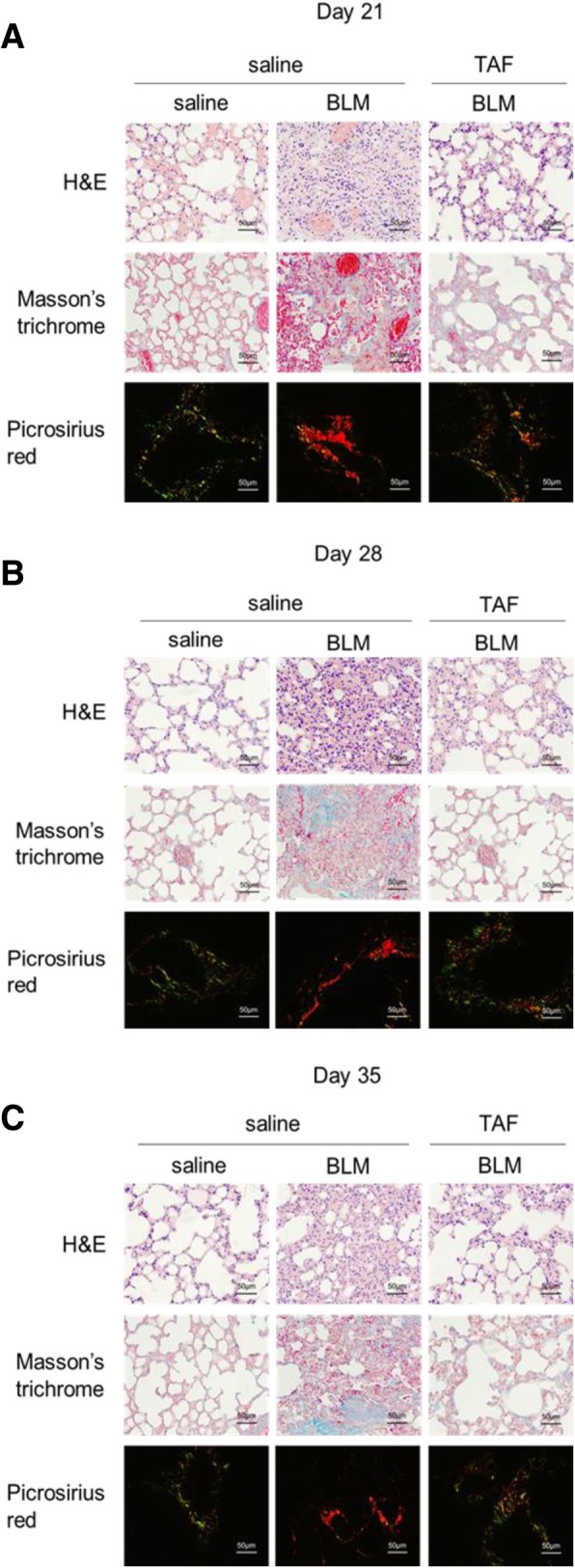
TAF attenuated BLM-induced lung fibrosis in mice. **a**-**c** Paraffin sections from lung tissues of the mice on day 21, day 28, or day 35 were stained with H&E, Masson’s trichrome staining or Picrosirius red stain. Left: in control group, alveolar structure was integrated and distinct, with no obvious exudation (H&E), a few collagen fibers (stained blue) in the alveolar walls were detected (Masson’s trichrome staining), and the type of fibers were type III collagen fibers (stained green); middle: in BLM group, significant lung consolidation, alveolar septum thickness and vascular congestion were detected, with a large amount of collagen deposition in the alveolar walls, and the type of fibers were type I collagen fibers (stained yellow and orange); right: in TAF group, histological changes were intermediate between the control group and BLM group. Original magnification of photomicrographs: × 400 (scale bar = 50 μm)

Paraffin sections from left lungs were stained hematoxylin and eosin and used to assess the histopathological changes in different groups. In the control group (saline + saline), no obvious histological changes were observed in the lung tissues, while significant increases in lung consolidation, alveolar septum thickness, inflammatory cell infiltration, vascular congestion, and alveolar collapse were detected in the BLM group (BLM + saline); changes in TAF group (BLM + TAF) were intermediate between the control group and BLM group (Fig. 3a–c). To further investigate whether TAF improves lung fibrosis, we used Masson’s trichrome staining to evaluate the degree of collagen deposition in the lung tissues. A few collagen fibers (stained blue) in the alveolar walls were detected in control group and TAF group and a large amount of collagen deposition and fibrotic areas was detected in the BLM + saline group (Fig. 3a–c). Accordingly, the Ashcroft score and collagen fiber area in each group were in accordance with the change in histopathological changes (Additional file 1: Table S2.1, Table S2.2). Additionally, picrosirius red staining was conducted to evaluate the collagen density, deposition pattern, and maturity. By using polarized light microscopy under a condition of birefringence pattern and hue, we found that in the control group, only a few green type III collagen fibers were distributed in the pulmonary alveoli; The distribution of collagen was similar to that in the control group but with a few more yellow type I collagen fibers. In the BLM group, thick yellow and orange I collagen fibers were significantly increased in the pulmonary alveoli and around the blood vessels, with some type III collagen fibers present.

### TAF reduces the expression of TGF-β1, collagen 3α1, α-SMA, and fibronectin in lung tissues of BLM-treated mice

**Fig. 4:**
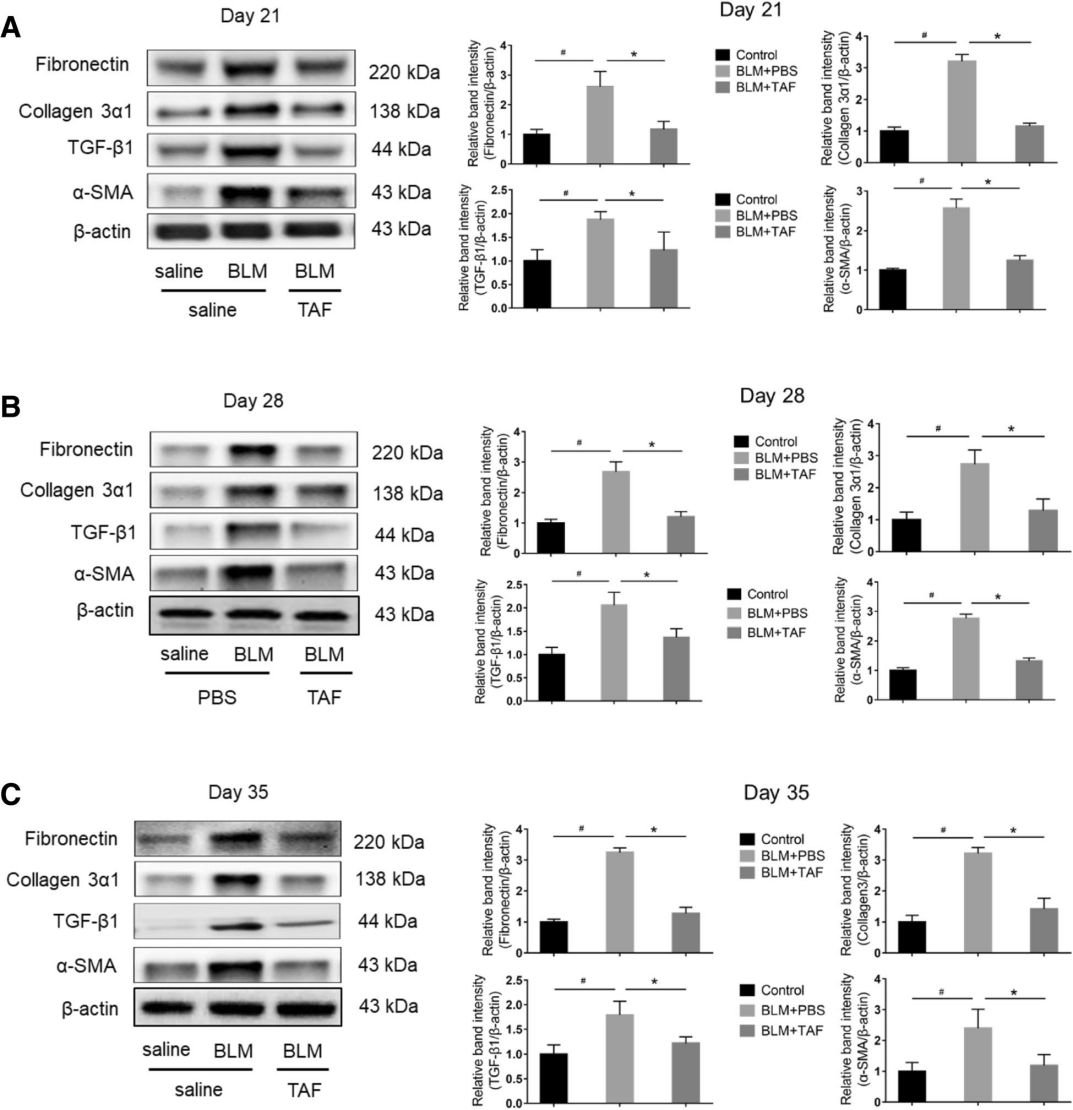
TAF suppressed TGF-β1, collagen 3α1, α-SMA, and fibronectin expression in lung tissues of BLM-treated mice. **a** Western blot analysis of TGF-β1, collagen 3α1, α-SMA, and fibronectin expression in lung tissues of mice sacrificed on day 21 after administration of saline or BLM; (**b**) The expression level of the above-mentioned production on day 28; (**c**) The expression level of the above-mentioned production on day 35. Increased protein levels of the above-mentioned targets were observed in BLM group (BLM + saline) compared with control group (saline + saline) and TAF group (BLM + TAF). All data were analyzed by densitometry and normalized to β-actin. Data were presented as mean ± standard error of the mean (SEM) of at least three separate experiments, and statistical significance across intervention groups was determined using one-way ANOVA. #*P* < 0.05 vs. control group. *P < 0.05 vs. BLM group

TGF-β1 is a profibrotic cytokine that mediates the development of pulmonary fibrosis induced by BLM [12, 27]. Additionally, the activation and proliferation of myofibroblasts are important signs of lung and liver fibrosis [37]. Increased expression of α-SMA and fibronectin have been detected in fibroblasts induced by the pro-fibrogenic cytokine [29]. Thus, α-SMA and fibronectin represent key markers of myofibroblast differentiation. Moreover, excessive deposition of collagen also reflects the severity of fibrosis [32]. To assess the effects of TAF on TGF-β1 expression, we detected the expression level of TGF-β1 in murine lung tissues by western blotting. Our results showed that in groups of mice treated on day 21, the expression of TGF-β1, collagen 3α1, α-SMA, and fibronectin was significantly reduced in TAF group compared to in the BLM group (*P* < 0.05) (Fig. 4a, b). The results on days 28 and 35 were consistent with those on day 21 (Fig. 4c).

### TAF upregulated the expression of NS5ATP9 at the RNA level in BLM-induced mice

**Fig. 5:**
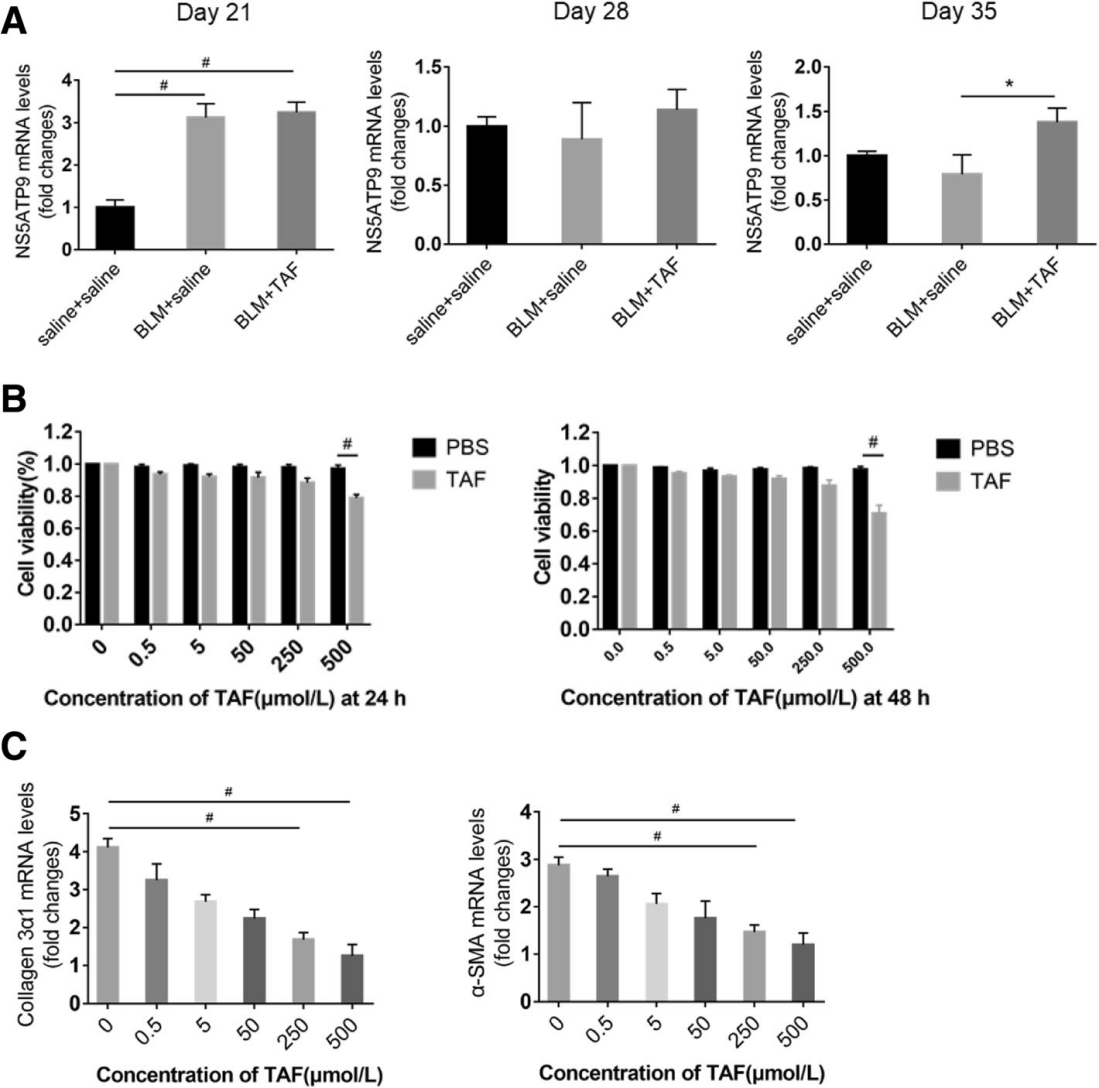
NS5ATP9 mRNA level in lung tissues of BLM-induced mice at different time points and the optimum concentration of TAF on cells. **a** NS5ATP9 RNA level in mice sacrificed on day 21, day 28 and day 35. Mice were respectively sacrificed on 21st, 28th, and 35th days, after 3 weeks under different intervention conditions of TAF or PBS separately from day 0, day 7, or day 14; then, the left lung section was excised and lysed, and used for mRNA levels of NS5ATP9. NS5ATP9 was determined by RT-qPCR. **b** Cells were treated with various concentrations (0.5, 5, 50, 250, 500 μmol/L) of TAF for 24 h or 48 h, and cell viability was measured using the CCK-8 assay. **c** Cells were serum-starved overnight, before incubation with TGF-β1 (5 ng/ml) or vehicle for 24 h, and then treated with vehicle or TAF with the concentrations of 0.5, 5, 50, 250, and 500 μmol/L. Besides, 24 h after compound dosing, the cells were collected and lysed, and used for detecting mRNA levels of α-SMA and collagen 3α1. Collagen 3α1 and TGF-β1 were determined by RT-qPCR. Statistical significance across intervention groups was determined using one-way ANOVA. #*P* < 0.05 vs. control group or untreated cells. *P < 0.05 vs. BLM group

We evaluated the relationship between the anti-fibrotic effect of TAF and negative feedback effect of NS5ATP9. We further examined the RNA level of NS5ATP9 in mice by RT-qPCR. We found that NS5ATP9 was significantly upregulated both in the BLM + TAF group and BLM + saline group compared to in the control group in mice sacrificed on day 21. In mice sacrificed on day 28, the expression level of NS5ATP9 was downregulated in both the BLM + TAF group and BLM + saline group; however, the downregulated trend in the BLM + saline group was greater than that in the BLM + TAF group. Furthermore, in mice sacrificed on day 35, the expression level of NS5ATP9 in the BLM + saline group was still downregulated, whereas the expression level of NS5ATP9 in the BLM + TAF group was upregulated; the difference between the two groups was significant (Fig. 5a).

### TAF inhibits TGF-β1-induced phosphorylation of Smad3 in lung fibroblasts

We investigated whether TAF is involved in the TGF-β1/Smad3 signaling pathway. We first examined the effect of TAF on the viability of lung fibroblasts. TGF-β1-stimulated cells were treated with vehicle or TAF at concentrations of 0.5, 5, 50, 250, and 500 μmol/L for 24 or 48 h, and cell viability was assessed by using CCK-8 assay. As illustrated in Fig. 5b, TAF at 500 μmol/L significantly reduced cell viability after 24 h or 48 h of incubation compared to control group. Then the effects of TAF on cells were examined by mRNA expression of α-SMA and collagen 3α1, and we found their expression significantly decreased by TAF at 250 μmol/L and 500 μmol/L (Fig. 5c). As a consequence, we determined 250 μmol/L as the optimum concentration of TAF according the experimental data.

**Fig. 6:**
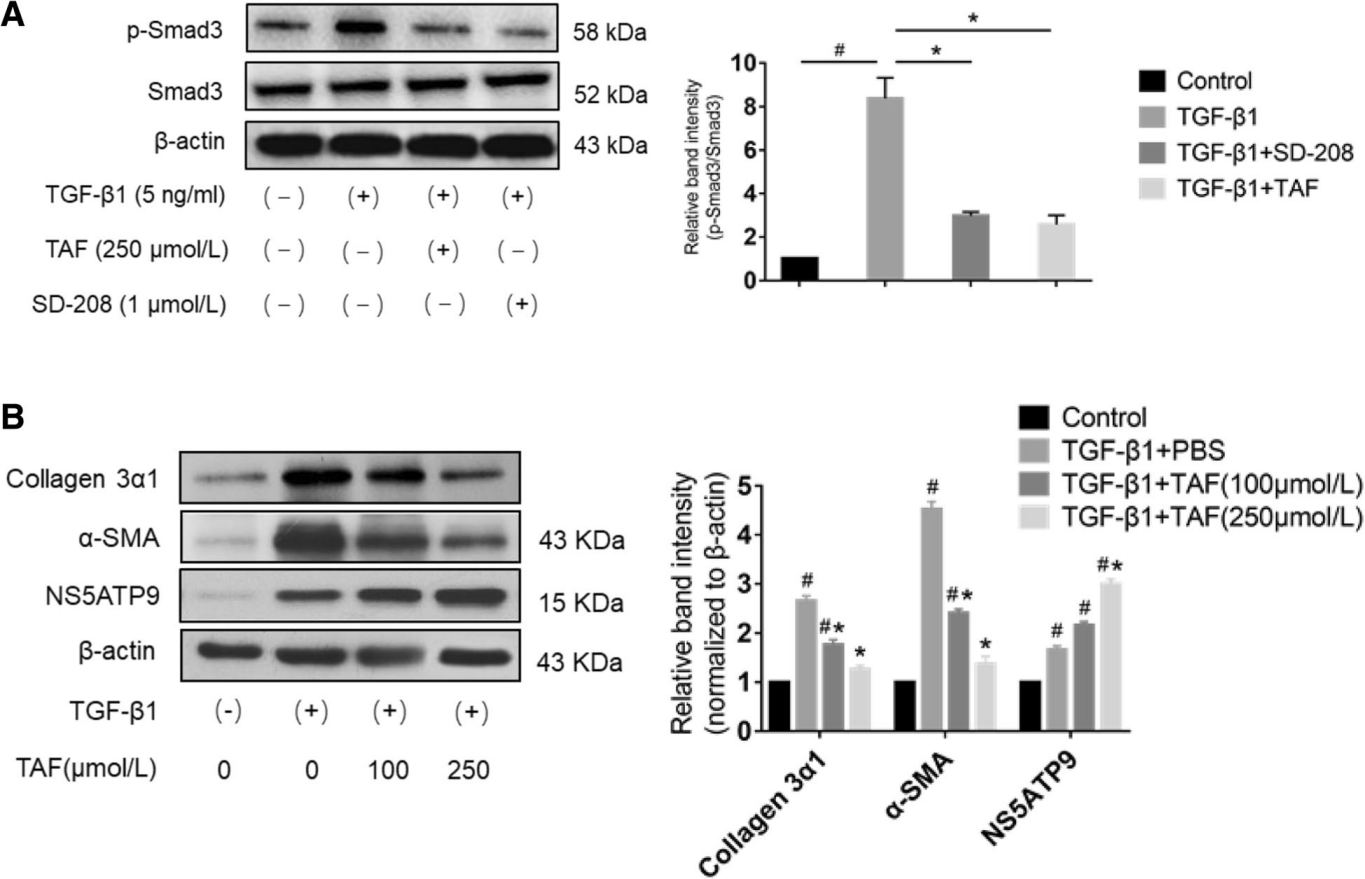
TAF decreased the expression level of phosphorylated-Smad3 and down-regulated α-SMA and collagen 3α1 by up-regulating NS5ATP9 expression in activated HFL1 cells. **a** Protein expression of phosphorylated-Smad3 and Smad3 under different intervention conditions. Cells were serum-starved overnight, before incubation with TGF-β1 (5 ng/ml) for 24 h, and then treated with SD-208 (1 μmol/L) or TAF (250 μmol/L) for 24 h. Phosphorylated and total Smad3 were measured by Western blotting against phosphorylated-Smad3 and Smad3. Blots were analyzed by densitometry and the results were expressed as relative units. All data were demonstrated as fold changes of gene expression normalized to β-actin. **b** Protein expression of α-SMA, collagen 3α1 and NS5ATP9 in cells stimulated by TGF-β1. Cells were serum-starved overnight before activated with TGF-β1 (5 ng/ml) for 24 h. Finally, cells were collected and lysed, and used for protein levels. All the objectives were determined by Western blotting. Blots of collagen 3α1, α-SMA and NS5ATP9 were analyzed by densitometry, and the results were expressed as relative units. Data were presented as mean ± standard error of the mean (SEM) of three independent trials performed in duplicate, and statistical significance across intervention groups was determined using one-way ANOVA. #*P* < 0.05 vs. untreated cells. *P < 0.05 vs. control (TGF-β1-treated) cells

Moreover, SD-208, a selective TGF-βRI (ALK5) kinase inhibitor that suppresses TGF-β-evoked cell growth, migration, and invasion, was used to assess the suppression of Smad3 and phosphorylated Smad3 [38]. Serum-starved HFL1 cells were treated with vehicle, SD-208 (1 μmol/L) or TAF (250 μmol/L) for 24 h following TGF-β1 stimulation (5 ng/mL) or vehicle treatment for 24 h. TGF-β1 upregulated the expression level of phosphorylated Smad3 in HFL1 cells, while TAF significantly decreased the expression level of phosphorylated-Smad3 in TGF-β1-treated cells, which supported the effect of SD-208, whereas the total expression level of Smad3 in each group remained consistent (Fig. 6a). These results suggest that TAF is involved in regulating the TGF-β1/Smad3 signaling pathway.

### TAF upregulates the expression level of NS5ATP9

In previous studies, we verified the negative feedback regulatory role of NS5ATP9 as well as anti-fibrotic effect and one of the signaling pathways of TAF. Based on the results, we further investigated the relationships among NS5ATP9, TAF, and the TGF-β1/Smad3 pathway. HFL1 cells were stimulated with human recombinant TGF-β1 prior to being treated with 250 μmol/L TAF for 24 or 48 h. The control group was treated with vehicle. The protein level of NS5ATP9 was markedly enhanced after TAF treatment in a time-dependent manner, while a decrease in collagen 3α1 and α-SMA were also detected (Fig. 6b).

## Discussion

The ideal animal model of pulmonary fibrosis must be able to replicate the histological characteristics of the typical common type of interstitial pneumonia. Bleomycin is the most commonly used drug to induce pulmonary fibrosis due to its low price and high repeatability. C57BL/6 mice are most sensitive to bleomycin and are currently the most commonly used experimental animal for pulmonary fibrosis. At present, single dose intratracheal infusion of bleomycin is the most commonly used method. However, studies have shown that lesions of mice in this model is self-limited after 6 weeks of administration [39]. What’s more, it is reported that bleomycin-induced lung injury in mice involves two phases. The first phase takes place over the first week after BLM administration, which is characterized by accumulation of inflammation mediators and inflammatory cells in the parenchyma and airways. The second phase starts from the second week, with the beginning of fibrogenesis [6]. So that in this study we tried to investigate the effect of TAF on early stage of pulmonary fibrosis.

The gene for NS5ATP9, is located at 15q22.1, is 336 base pairs and encodes a protein containing 111 amino acids. Using a yeast two-hybrid assay, the gene product of NS5ATP9 was originally defined as a proliferating cell nuclear antigen binding protein [18]. Shi et al. identified NS5ATP9 as a trans-activated gene of hepatitis C virus of NS5A protein by suppression subtractive hybridization [16]. Zhang et al. demonstrated the inhibition effect of NS5ATP9 on TGF-β1-induced hepatic stellate cell activation and proliferation [14]. In this study, we found an opposite change between NS5ATP9 and fibrosis-related factors (collagen 3α1, α-SMA, fibronectin) in TGF-β1-induced HFL1 cells, suggesting that NS5ATP9 protects against the pathogenesis and progression of pulmonary fibrosis.

Chan et al. reported that TAF improved liver inflammation and fibrosis in patients with HBV [27]. In our prior experiment, TAF showed anti-inflammatory and anti-fibrotic efficacy in a mouse model of CCl_4_-induced liver fibrosis and hepatic stellate cells compared to other nucleotide analogues (data unpublished). As the optimal dose of TAF in patients infected HBV is 25 mg per day, we tried to investigate the effect of it on pulmonary fibrosis by using the same dose, which was converted into mice dosage (5.125 mg/kg) according to the conversion formulas for humans and animals before given in our animal experiment. In this study, TAF showed strong anti-fibrotic properties by significantly improving pathological changes and decreasing biochemical fibrotic parameters. In recent years, although two drugs, pirfenidone and nintedanib, showed effects in clinical trials, their actual efficacies are low. Our results may be useful for developing new therapeutic approaches for pulmonary fibrosis. Additionally, in vitro, we found that TAF reduced the expression of phosphorylated-Smad3 protein levels and inhibited TAF on TGF-β1/Smad3 signaling in accordance with SD-208, suggesting that TAF is involved in regulating the TGF-β1/Smad3 signaling pathway.

Importantly, when comparing the improvement of mortality and body weight loss among the three TAF-treatment groups, we observed lower mortality rates and reduced weight loss in the group administered TAF on day 0 after administration of BLM. As the greatest inflammatory response was within 2 weeks in BLM-induced mice, we predicted that TAF may also alleviate fibrosis and mortality by suppressing inflammation (inhibition of NLRP3, data not published), but this hypothesis requires further analysis.

In the animal experiment, we found an increased trend of NS5ATP9 in the TAF treatment group. However, interestingly, after 3 weeks of treatment, NS5ATP9 expression was enhanced in mice treated with TAF starting on day 0 but weakened in mice treated with TAF from day 7, and increased again in mice treated with TAF from day 14. Therefore, TAF inhibited pulmonary fibrosis possibly by upregulating the expression level of NS5ATP9, and TAF may be involved in depleting NS5ATP9. First, the expression level NS5ATP9 may be upregulated to inhibit fibrosis in the early stage of pulmonary fibrosis, followed by fibrosis progression, and NS5ATP9 is gradually depleted as its expression level is downregulated. TAF then exerts its function by increasing the expression level of NS5ATP9. However, because of the lack of dynamic changes of NS5ATP9 in different periods in our experimental design, additional data are needed to support our hypothesis. By comparing the Ashcroft score between the TAF group and BLM group, we observed a higher expression level of NS5ATP9 and less severe pulmonary fibrosis. Additionally, in our primary study (unpublished data), when we treated mice with different doses of BLM (2 mg/kg, 2.5 mg/kg and 5 mg/kg), we found higher RNA expression of NS5ATP9 in BLM treated groups compared to control group, and higher RNA level of NS5ATP9 in group with lower dose of BLM (See Additional file 1: Figure S3). These results suggest that NS5ATP9 is associated with lung injury and can be used to evaluate the severity and drug treatment effects in lung fibrosis.

Furthermore, our in vitro study confirmed the relationship between TAF and NS5ATP9. We found NS5ATP9 was increased by TAF; over time, the drug increased the expression of NS5ATP9. The results suggest that NS5ATP9 can be used as a therapeutic target, and drugs that promotes its expression may be useful for treating pulmonary fibrosis. We also found that the inhibitory action of TAF on the TGF-β1/Smad3 pathway resulted from enhancement of NS5ATP9, suggesting that NS5ATP9 plays an important role in pulmonary fibrosis. Whether these effects occur upstream or downstream of TGF-β1 must be further studied.

## Conclusion

**Fig. 7:**
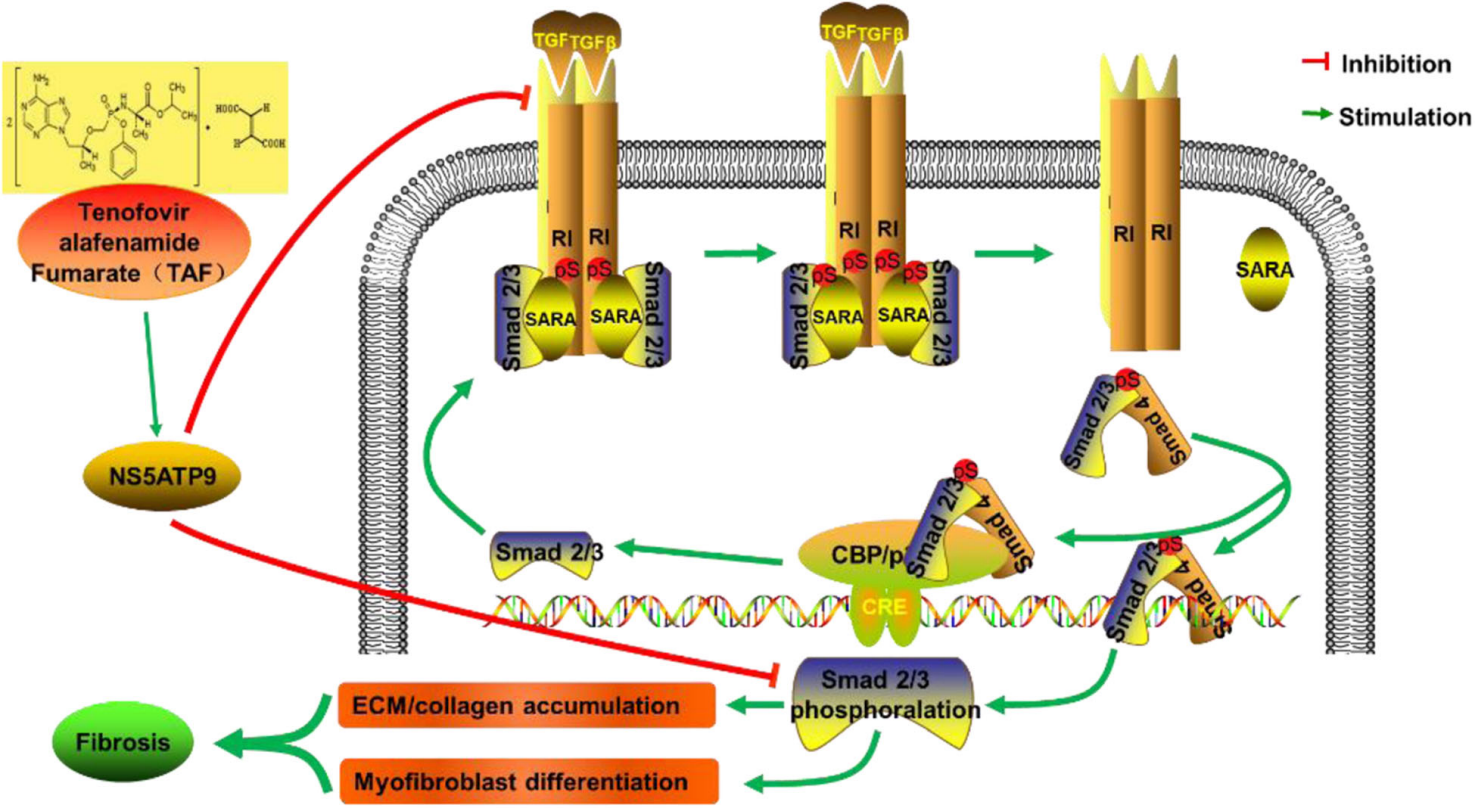
Illustration of the anti-fibrotic effects and their relationship between TAF and NS5ATP9 in BLM-induced pulmonary fibrosis. By originally up-regulating the expression level of NS5ATP9, TAF suppresses BLM-induced lung fibrosis via inhibition of differentiation of fibroblast into myofibroblast and ECM production, as well as down-regulating the expression level of collagen 3α1, fibronectin, and αSMA, in addition to TGF-β1/Smad3 signaling pathway

In conclusion, our findings may extend the functions of NS5ATP9 and medication indications of TAF, reflecting that the NS5ATP9 is a gene product associated with negative feedback regulation in lung fibrosis and is closely associated with TAF (Fig. 7). By upregulating the expression level of NS5ATP9, TAF efficiently improved BLM-induced lung fibrosis by inhibiting the differentiation of fibroblasts into myofibroblasts and increasing ECM production as well as the expression of phosphorylated-Smad3, among other proteins. These results suggest that TAF can be used to prevent and treat pulmonary fibrosis and that NS5ATP9 should be evaluated as a target gene for drug selection and biomarker for evaluating the therapeutic effect and prognosis in the treatment of lung fibrosis.

## Additional file

Additional file 1: **Table S1.1.** Human Primers Used for conducting RT-qPCR. **Table S1.2** Mouse Primers Used for performing RT-qPCR. **Table S2.1.** Ashcroft scores in each group (*n* = 5, ^−^x + s). **Table S2.2.** The collagen fiber area in each group. **Table S3.** Antibodies used in Western blot. **Figure S1.** picrosirius red staining. **Figure S2.** Grouping details. **Figure S3.** The expression of TGF-β1, α-SMA and NS5ATP9 under different dose of bleomycin in mice. (DOCX 12948 kb)

## Data Availability

The animal model we used was a classical bleomycin-induced pulmonary fibrosis mouse model. And the experiment techniques we used such as RT-qPCR, Western blot and hematoxylin and eosin staining were according to related standard protocols.

## Abbreviations

BLM: Bleomycin
Collagen 3α1: Collagen type 3 (α 1 chain)
ECM: Extracellular matrix
NS5ATP9: Non-structural protein NS5A transactivated protein 9 of hepatitis C virus
TAF: Tenofovir alafenamide fumarate
TGF-β1: Transforming growth factor beta 1
α-SMA: α-smooth muscle actin

## References

[1] Fernandez Perez ER, Daniels CE, Schroeder DR (2010). Incidence, prevalence, and clinical course of idiopathic pulmonary fibrosis: a population-based study. Chest.

[2] Richeldi L, Collard HR, Jones MG (2017). Idiopathic pulmonary fibrosis. Lancet.

[3] Weiss RB, Muggia FM (1980). Cytotoxic drug-induced pulmonary disease: update 1980. Am J Med.

[4] Moeller A, Ask K, Warburton D (2008). The bleomycin animal model: a useful tool to investigate treatment options for idiopathic pulmonary fibrosis?. Int J Biochem Cell Biol.

[5] Fattman CL, Tan RJ, Tobolewski JM (2006). Increased sensitivity to asbestos-induced lung injury in mice lacking extracellular superoxide dismutase. Free Radic Biol Med.

[6] Moore BB, Hogaboam CM (2008). Murine models of pulmonary fibrosis. Am J Physiol Lung Cell Mol Physiol.

[7] Hoozen BEV, Grimmer KL, Marelich GP (2000). Early phase collagen synthesis in lungs of rats exposed to bleomycin. Toxicology.

[8] Majamaa K, Jokinen A, Stolle C (1993). Exclusion of mutations in the gene for type III collagen (COL3A1) as a common cause of intracranial aneurysms or cervical artery dissections: results from sequence analysis of the coding sequences of type III collagen from 55 unrelated patients. Neurology.

[9] Raghu G, Striker LJ, Hudson LD (1985). Extracellular matrix in normal and fibrotic human lungs. Am Rev Respir Dis.

[10] Ley B, Brown KK, Collard HR (2014). Molecular biomarkers in idiopathic pulmonary fibrosis. Am J Physiol Lung Cell Mol Physiol.

[11] Dennler S, Goumans MJ, Dijke P (2002). Transforming growth factor beta signal transduction. J Leukoc Biol.

[12] Fernandez IE, Eickelberg O (2012). The impact of TGF-β on lung fibrosis: from targeting to biomarkers. Proc Am Thorac Soc.

[13] Shi K, Jiang J, Ma T (2014). Dexamethasone attenuates bleomycin-induced lung fibrosis in mice through TGF-β, Smad3 and JAK-STAT pathway. Int J Clin Exp Med.

[14] Flanders KC (2004). Smad3 as a mediator of the fibrotic response. Int J Exp Pathol.

[15] Ahn J, Kim M, Lim M (2011). The inhibitory effect of ginsan on TGF-β mediated fibrotic process. J Cell Physiol.

[16] Shi L, Zhang SL, Li K (2008). NS5ATP9, a gene up-regulated by HCV NS5A protein. Cancer Lett.

[17] Quan M, Liu S, Li G (2014). A functional role for NS5ATP9 in the induction of HCV NS5A-mediated autophagy. J Viral Hepat.

[18] Wang Q, Wang Y, Li Y (2013). NS5ATP9 contributes to inhibition of cell proliferation by hepatitis C virus (HCV) nonstructural protein 5A (NS5A) via MEK/ extracellular signal regulated kinase (ERK) pathway. Int J Mol Sci.

[19] Yu P, Huang B, Shen M (2001). P15 (PAF), a novel PCNA associated factor with increased expression in tumor tissues. Oncogene.

[20] Mizutani K, Onda M, Asaka S (2005). Overexpressed in anaplastic thyroid carcinoma-1 (OEATC-1) as a novel gene responsible for anaplastic thyroid carcinoma. Cancer.

[21] Petroziello J, Yamane A, Westendorf L (2004). Suppression subtractive hybridization and expression profiling identifies a unique set of genes overexpressed in non-small-cell lung cancer. Oncogene.

[22] Yuan RH, Jeng YM, Pan HW (2007). Overexpression of KIAA0101 predicts high stage, early tumor recurrence, and poor prognosis of hepatocellular carcinoma. Clin Cancer Res.

[23] Zhu K, Diao D, Dang C (2013). Elevated KIAA0101 expression is a marker of recurrence in human gastric cancer. Cancer Sci.

[24] Kato T, Daigo Y, Aragaki M (2012). Overexpression of KIAA0101 predicts poor prognosis in primary lung cancer patients. Lung Cancer.

[25] Zhang M, Zhang J, Liu S (2015). NS5ATP9 suppresses activation of human hepatic stellate cells, possibly via inhibition of Smad3/phosphorylated-Smad3 expression. Inflammation.

[26] Sax PE, Zolopa A, Brar I (2014). Tenofovir alafenamide vs. tenofovir disoproxil fumarate in single tablet regimen s for initial HIV-1 therapy: a randomized phase 2 study. J Acquir Immune Defic Syndr.

[27] Chan HL, Fung S, Seto WK (2016). Tenofovir alafenamide versus tenofovir disoproxil fumarate for the treatment of HBeAg-positive chronic hepatitis B virus infection: a randomised, double-blind, phase 3, non-inferiority trial. Lancet Gastroenterol Hepatol.

[28] Marcellin P, Gane E, Buti M (2013). Regression of cirrhosis during treatment with tenofovir disoproxil fumarate for chronic hepatitis b: a 5-year open-label follow-up study. Lancet.

[29] Feig JL, Mediero A, Corciulo C (2017). The antiviral drug tenofovir, an inhibitor of pannexin-1-mediated atp release, prevents liver and skin fibrosis by downregulating adenosine levels in the liver and skin. PLoS One.

[30] Longo DL, Rockey DC, Bell PD (2015). Fibrosis — a common pathway to organ injury and failure. N Engl J Med.

[31] Makarev E, Izumchenko E, Aihara F (2016). Common pathway signature in lung and liver fibrosis. Cell Cycle.

[32] Zeisberg Michael, Kalluri Raghu (2013). Cellular Mechanisms of Tissue Fibrosis. 1. Common and organ-specific mechanisms associated with tissue fibrosis. American Journal of Physiology-Cell Physiology.

[33] Hladik F, Burgener A, Ballweber L (2015). Mucosal effects of tenofovir 1% gel. Elife.

[34] Rodríguez García M, Patel MV, Shen ZY, Bodwell JE, Rossoll RM, Wira CR (2017). Tenofovir inhibits wound healing of epithelial cells and fibroblasts from the upper and lower human female reproductive tract. Sci Rep.

[35] Stray KM, Park Y, Babusis D, Callebaut C, Cihlar T, Ray AS (2017). Tenofovir alafenamide (TAF) does not deplete mitochondrial DNA in human t-cell lines at intracellular concentrations exceeding clinically relevant drug exposures. Antivir Res.

[36] Sriram N, Kalayarasan S, Sudhandiran G (2008). Enhancement of antioxidant defense system by epigallocatechin-3-gallate during bleomycin induced experimental pulmonary fibrosis. Biol Pharm Bull.

[37] Hinz B, Phan SH, Thannickal VJ (2007). The myofibroblast: one function, multiple origins. Am J Pathol.

[38] Leung SY, Niimi A, Noble A (2006). Effect of transforming growth factor-beta receptor I kinase inhibitor 2,4-disubstituted pteridine (SD-208) in chronic allergic airway inflammation and remodeling. J Pharmacol Exp Ther.

[39] Chung MP, Monick MM, Hamzeh NY (2003). Role of repeated lung injury and genetic background in bleomycin-induced fibrosis. Am J Respir Cell Mol Biol.

