# Supplementary material for: Tenofovir alafenamide fumarate attenuates bleomycin-induced pulmonary fibrosis by upregulating the NS5ATP9 and TGF-β1/Smad3 signaling pathway
Source: Respir Res. 2019 Jul 22;20:163. doi: 10.1186/s12931-019-1102-2 (PMC6647111; doi:10.1186/s12931-019-1102-2)
Supplement: Supplementary file 1 — Table S1.1. Human Primers Used for conducting RT-qPCR. Table S1.2 Mouse Primers Used for performing RT-qPCR. Table S2.1. Ashcroft scores in each group (n = 5, −x + s). Table S2.2. The collagen fiber area in each group. Table S3. Antibodies used in Western blot. Figure S1. picrosirius red staining. Figure S2. Grouping details. Figure S3. The expression of TGF-β1, α-SMA and NS5ATP9 under different dose of bleomycin in mice. (DOCX 12948 kb) [file 12931_2019_1102_MOESM1_ESM.docx]

**Table 1.1** **Human Primers Used for conducting RT-qPCR**

| Gnens | Sense (5′–3′) | Antisense (5′–3′) |
| --- | --- | --- |
| α-SMA  Collagen 3α1  Fibronectin  NS5ATP9  β-actin | gggaatgggacaaaaagaca  ctggaccccagggtcttc  tccctcggaacatcagaaac  ttggttcttccacctctgcc  catccgcaaagacctgtacgc | cttcaggggcaacacgaa  gaccatctgatccagggtttc  cagtgggagacctcgagaag  cactgcttcctgcctcttca  agtacttgcgctcaggaggag |

**Table 1.2** **Mouse Primers Used for performing RT-qPCR**

| Genes | Sense (5′–3′) | Antisense (5′–3′) |
| --- | --- | --- |
| α-SMA  Collagen 3α1  Fibronectin  NS5ATP9  TGF-β1  β-actin | gagactctcttccagccatctt  aaggctgcaagatggatgct  gcagtgaccaccattactg  gcccctaggaaggtgctt  gaagtggatccacgagcccaag  ctaaggccaaccgtgaaaag | tgatctccttctgcatcctgtc  gtgcttacgtgggacagtca  ggtagccagtgagctgaacac  gggttccctcctgcatactt  gctgcacttgcaggagcgcac  accagaggcatacagggaca |

**Table 2.1 Ashcroft scores in each group (n=5, ‾x + s)**

| Time | Ashcroft scores |
| --- | --- |
|  | Control Group BLM Group TAF Group |
| Day 21 0.53±0.23 3.32±0.88# 0.93±0.12 #*  Day 28 0.77±0.36 4.16±0.55# 1.13±0.28 #*  Day 35 0.60±0.17 3.68±0.50# 1.04±0.29#* | |

**Table 2.2 The collagen fiber area in each group**

| Time | Collagen Volume Fraction (CVF) % |
| --- | --- |
|  | Control Group BLM Group TAF Group |
| Day 21 17.95±3.20 27.75±2.99 # 19.72±3.08 #*  Day 28 16.24±2.35 29.15±2.52 # 21.63±2.69#*  Day 35 17.12±2.88 27.06±3.12 # 20.22±2.85#* | |

**Antibodies used in Western blot**

Cell lysates were subjected to denaturating SDS gel electrophoresis followed by electroblotting and incubation with either monoclonal Anti-α-SMA antibody (1:1000, sc-53015，Santa Cruz Biotechnology, USA), monoclonal Anti-COL-I (against α1 chain, 1:200, ab21286, Abcam, USA), monoclonal Anti-COL-Ⅲ (against α1 chain, 1:1000, ab7778, Abcam, USA), monoclonal Anti-Smsd3 antibody (1:1000, ab40854, Abcam, USA), monoclonal Anti-Smad3 (phospho S423 + S425) antibody (1:2000, ab52903, Abcam, USA), monoclonal Anti-P15(NS5ATP9) antibody ( 1:500, sc-390515, Santa Cruz, USA), or monoclonal Anti-β-actin antibody (1:5000, A2228, SIGMA, USA).

Fig. 1 picrosirius red staining

Control group (day 21)


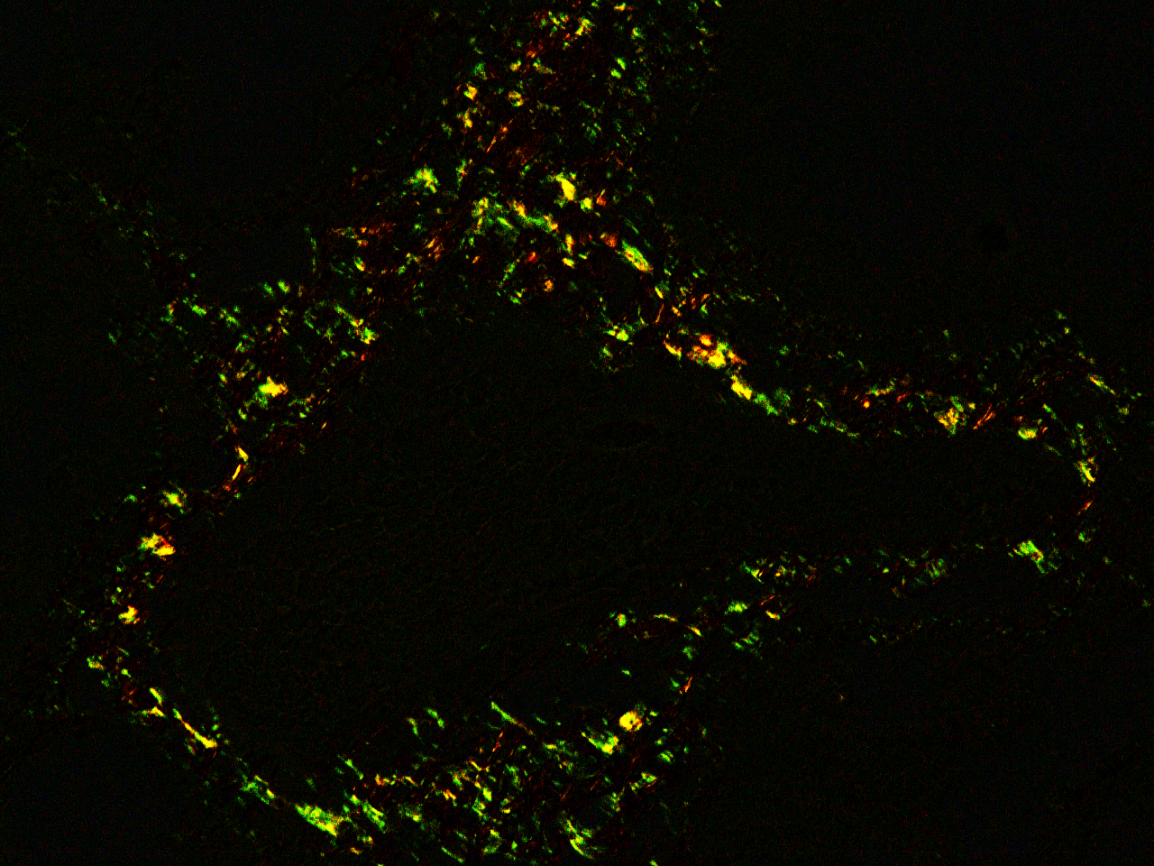


BLM group (day 21)


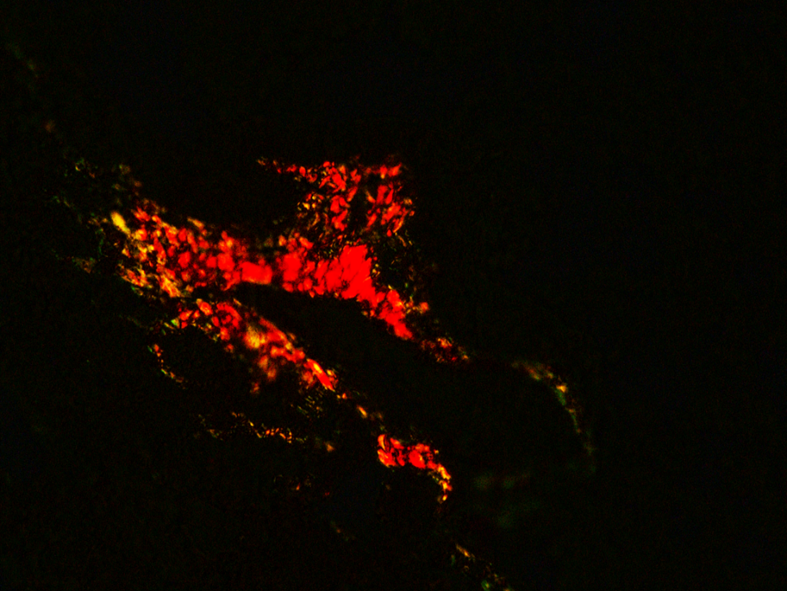


TAF group (day 21)


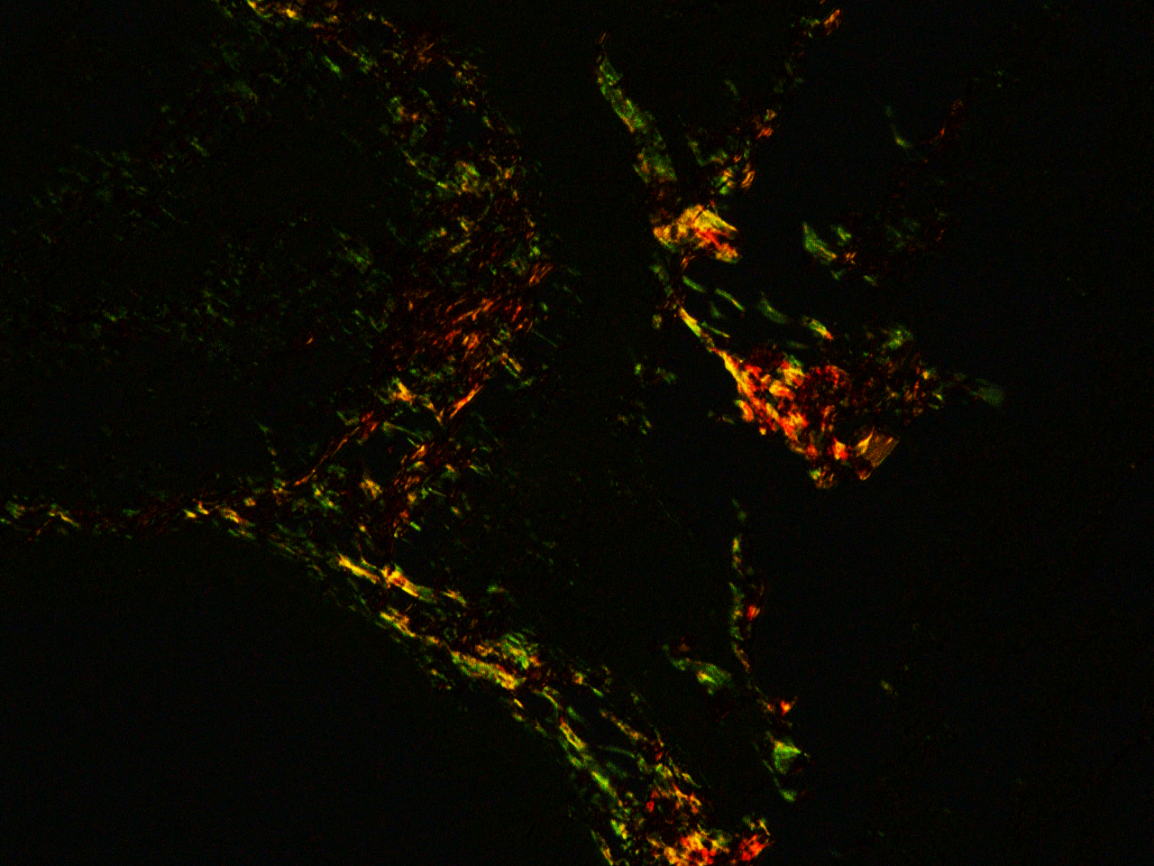


Control group (day 28)


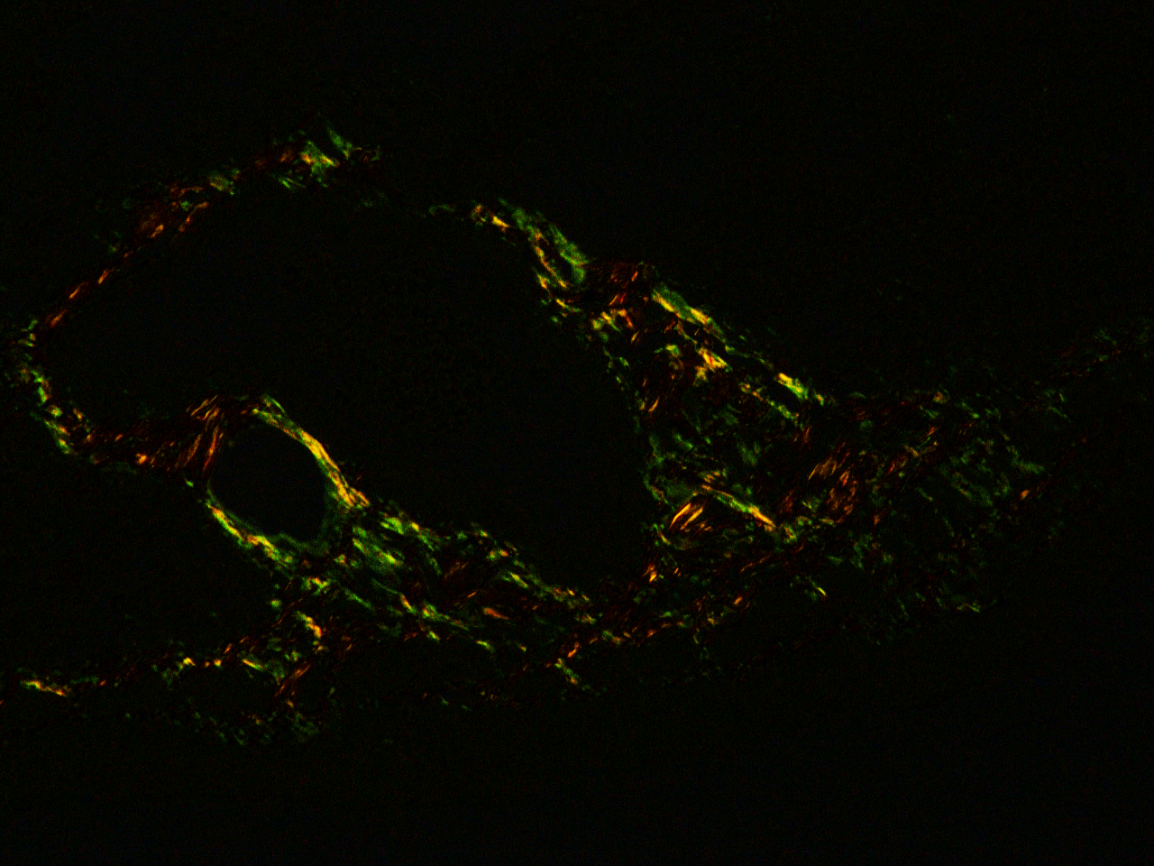


BLM group (day 28)


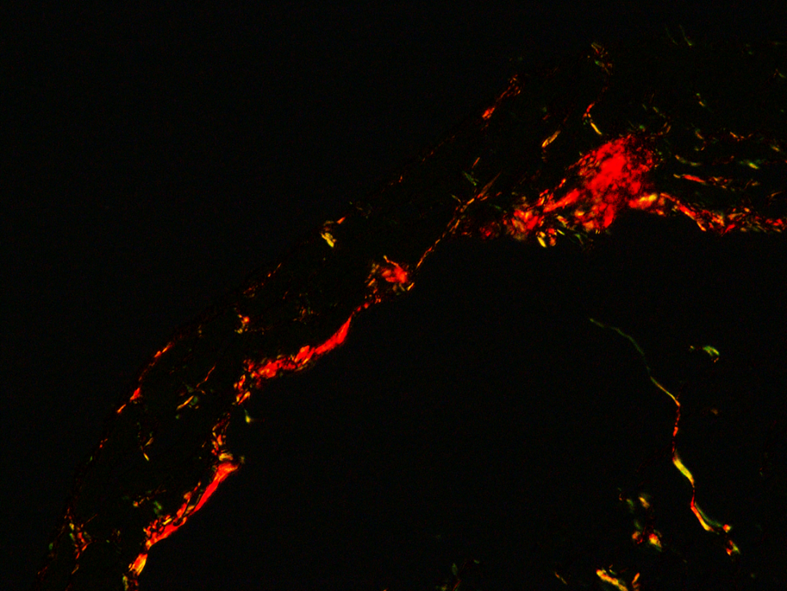


TAF group (day 28)


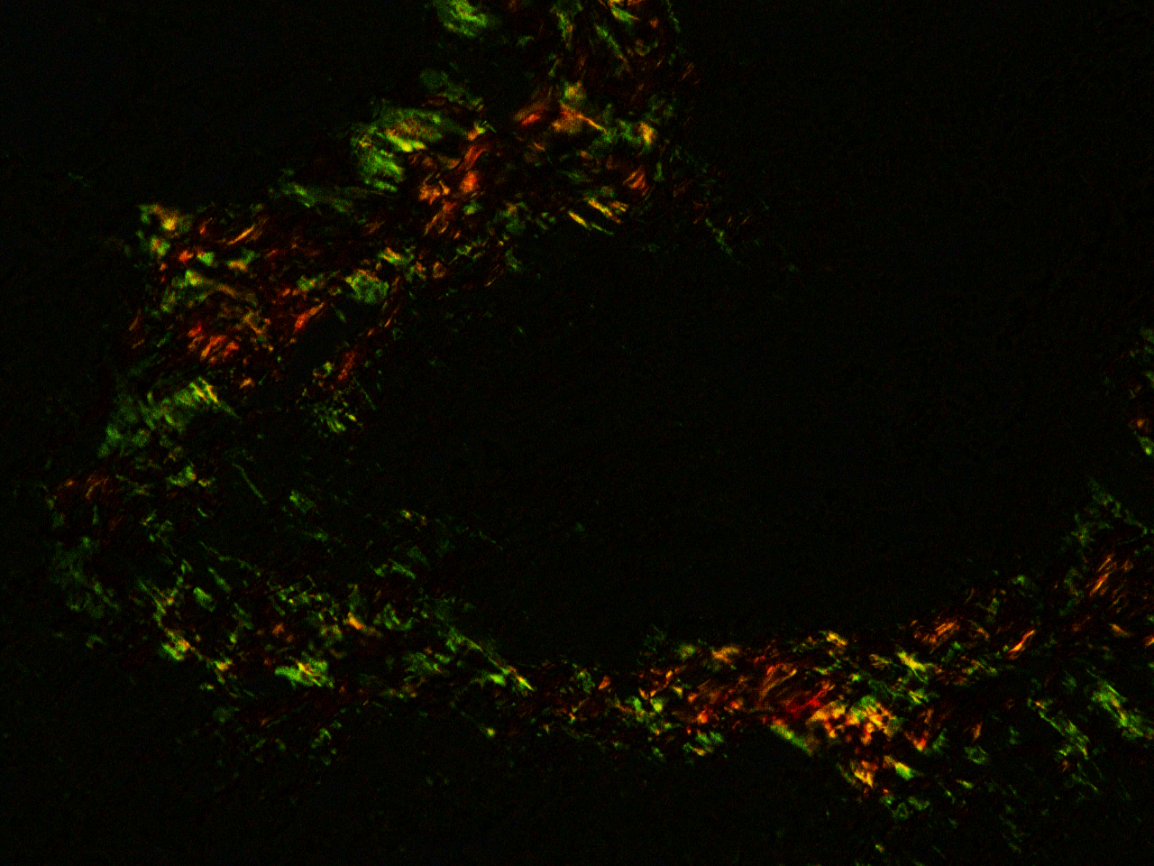


Control group (day 35)


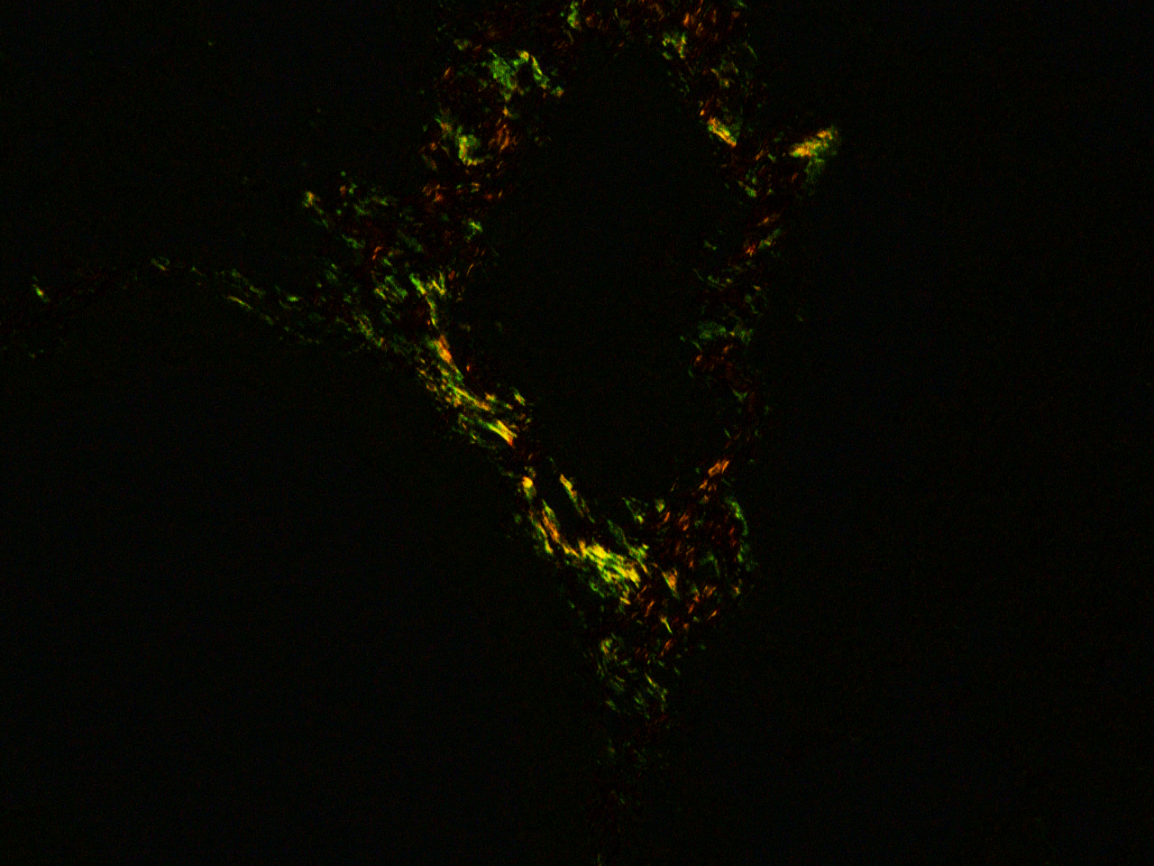


BLM group (day 35)


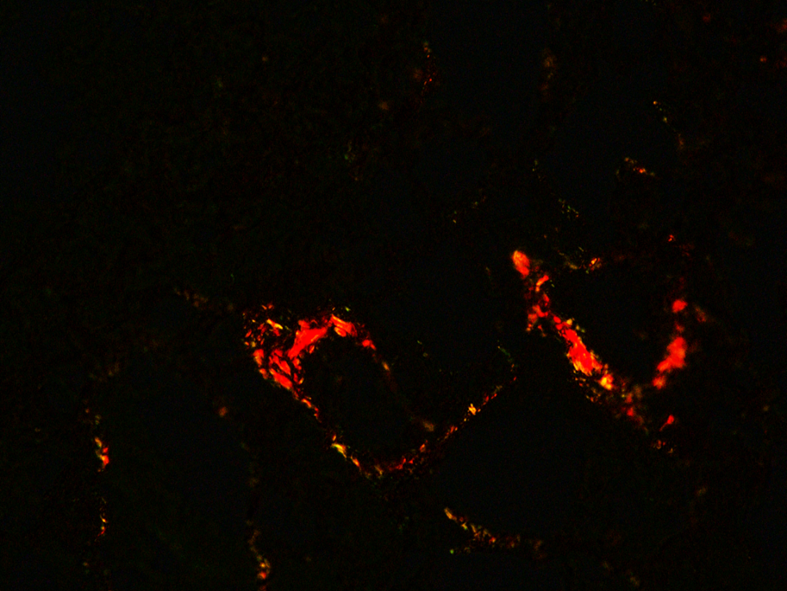


TAF group (day 35)


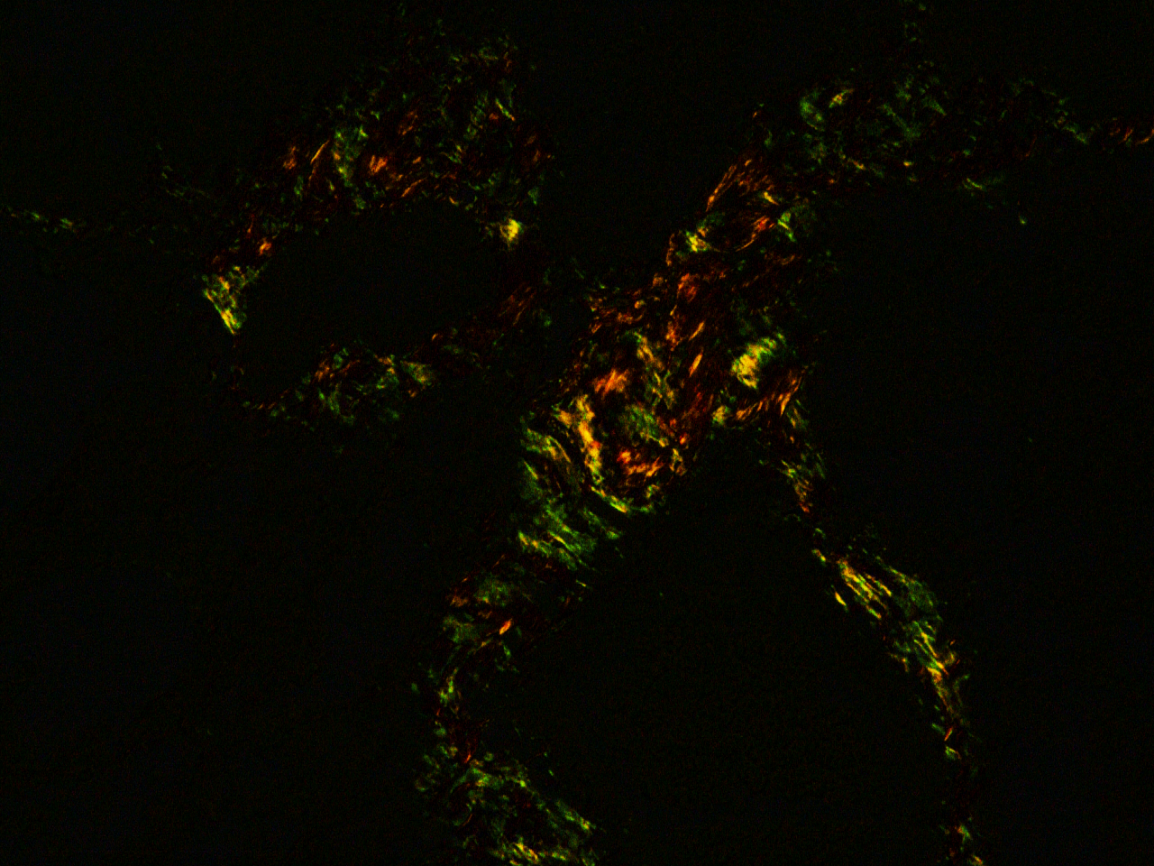


Fig. 2 Grouping details


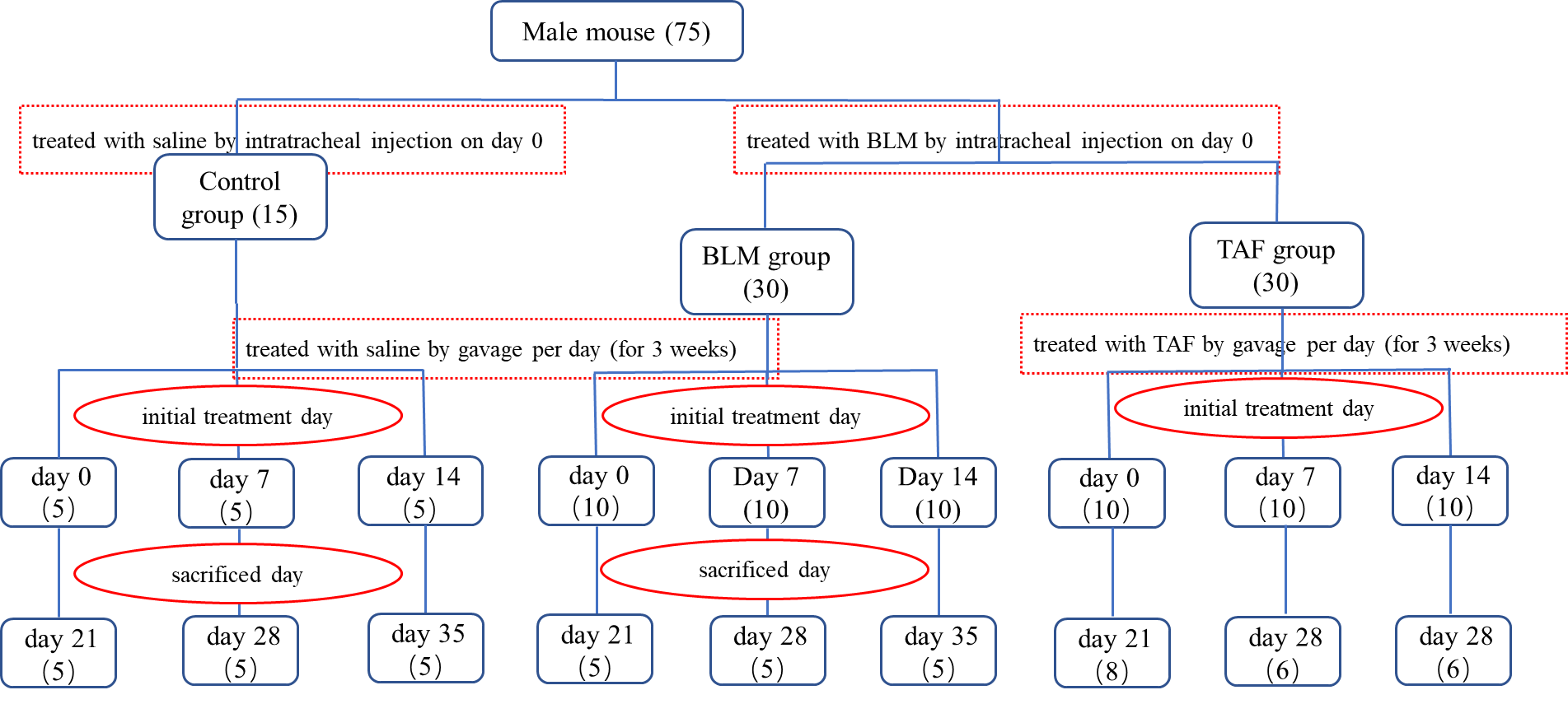


Fig. 3 The expression of TGF-β1, α-SMA and NS5ATP9 under different dose of bleomycin in mice


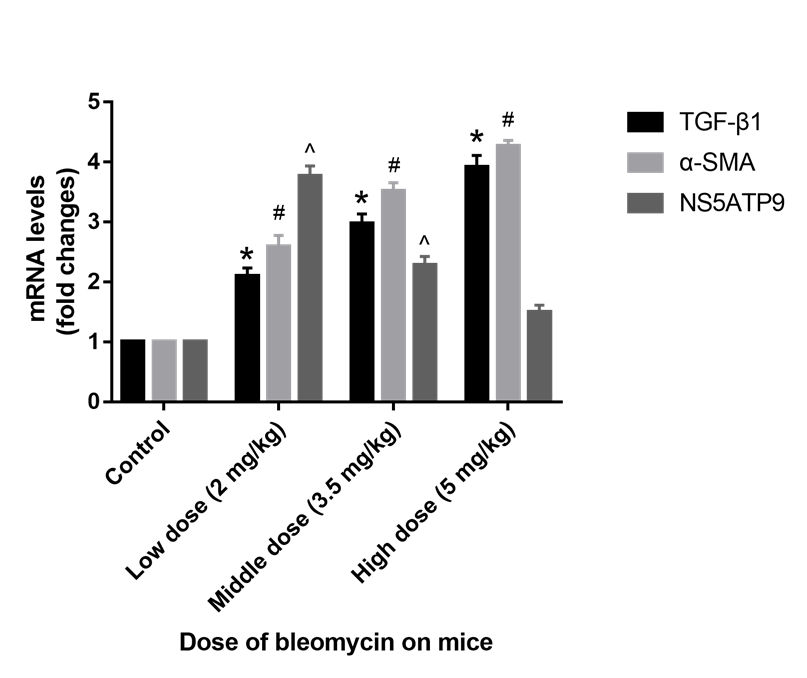


**P* < 0.05 vs. control group of TGF-β1, #*P* < 0.05 vs. control group of α-SMA, ^*P* vs. control group of NS5ATP9.
